# Giant Epicardial Cyst Eroding Left Ventricular Wall Mimicking as Simple Pericardial Cyst

**DOI:** 10.21470/1678-9741-2020-0623

**Published:** 2022

**Authors:** Kishore Gupta, Dhiren Shah, Dhaval Naik, Amit Chandan, Chintan Sheth, Gautam Shah

**Affiliations:** 1 Department of Cardiac Surgery, CIMS Hospital, Ahmedabad, Gujarat, India.; 2 Department of Pathology, CIMS Hospital, Ahmedabad,Gujarat, India.

**Keywords:** Pericardium, Mediastinal Cyst, Chest Pain, Myocardium, Coronary Vessels, Thoracotomy

## Abstract

Epicardial cysts are rarer benign tumors than pericardial cysts. There have been few reports on surgical management of epicardial cysts. A 17-year-old normotensive boy presented with chest pain and palpitations, which on evaluation was found to be a mediastinal mass (pericardial cyst). Surgical resection of the cyst via thoracotomy was planned. The cyst was diagnosed as an epicardial cyst intraoperatively. However, due to the epicardial origin of cyst and posterior adhesions, resection was done via midline approach. The base was formed by visceral pericardium and eroding into myocardium of left ventricle, so the resection was concluded with on-pump surgery. In case of erroneous diagnosis or undesirable finding, a safer midline approach with on-pump surgery, as an alternative to minimally invasive approach for complicated epicardial cysts (erosion into ventricle/lying in close proximity to important structures or near to coronary arteries) should be considered.

**Table:** 

Abbreviations, acronyms & symbols	
CPB	= Cardiopulmonary bypass
CT	= Computed tomography
LV	= Left ventricle
MRI	= Magnetic resonance imaging
STIR	= Short inversion time inversion recovery

## INTRODUCTION

We hereby report a case of a giant epicardial cyst eroding into the left ventricle which simulated a pericardial cyst on preoperative assessment.

### Case Report

**Fig. 1:**
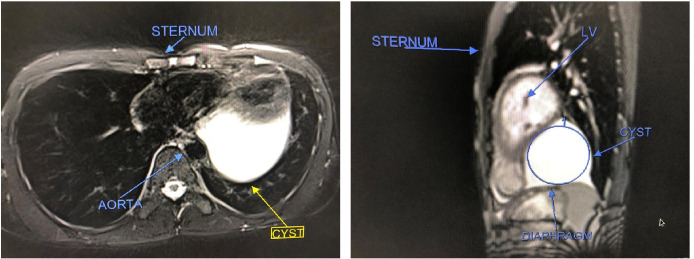
MRI in transverse section and sagittal view showing a well-defined cystic mass in the posterior and lateral portion abutting the left cardiac border.

A 17-year-old boy with no previous medical or surgical history presented with chest pain and off and on palpitations. Clinical examination revealed normal respiratory and cardiac examinations but elevated jugular venous pressure. Echocardiography revealed a large cyst (7×6.8×7 cm) around the posteroinferior part of mediastinum and extending laterally. Magnetic resonance imaging (MRI) revealed a well-defined cystic mass lesion in the posterior, inferior and lateral aspect of left cardiac border appearing hyperintense on short inversion time inversion recovery (STIR) sequences and hypointense on T1 sequence (Figure 1).

Therefore, a pericardial cyst removal via small thoracotomy was planned. Thoracotomy was done but pericardium was found to be normal, except adherent posteriorly and superiorly, and the cyst appeared to be epicardial in origin. Therefore, we planned to proceed via median sternotomy with cardiopulmonary bypass (CPB). The cyst was found to be pushing the ventricles anteriorly. In addition, the mass was adhered to the pericardium and the base was formed by visceral pericardium and myocardium of the inferior wall of the left ventricle. The cyst was most tightly attached to the left ventricle inferiorly. Thus, it was thought to have originated from the posteroinferior portion of the left ventricle (LV). Dissection of the mass was difficult because of adhesions and proximity to the posterior descending artery.

**Fig. 2:**
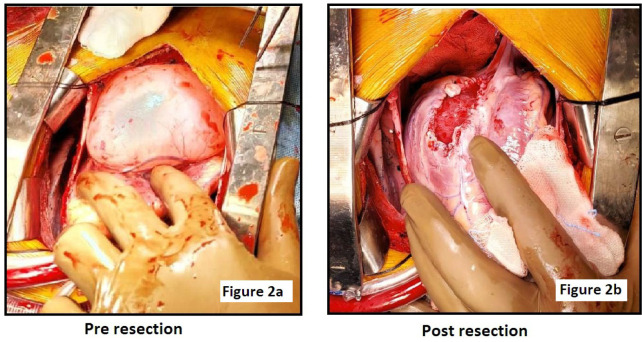
(A)Intraoperative image depicting large size of cyst and feeder vessels over the surface.(B)Postoperative image showing cyst floor with erosion of myocardial tissue and plicated edges.

Cyst wall was thick and with feeder vessels over the surface (Figure 2A). It was completely dissected and excised near its attachment. Inspection of the floor showed certain erosion of myocardial tissue, and the thickness of ventricular wall was attenuated. The cyst was trimmed, and multiple bleeding points along the cut edges were controlled with suture ligatures. The edges were then plicated over the attenuated ventricular wall with sutures to reinforce it (Figure 2B).

**Fig. 3:**
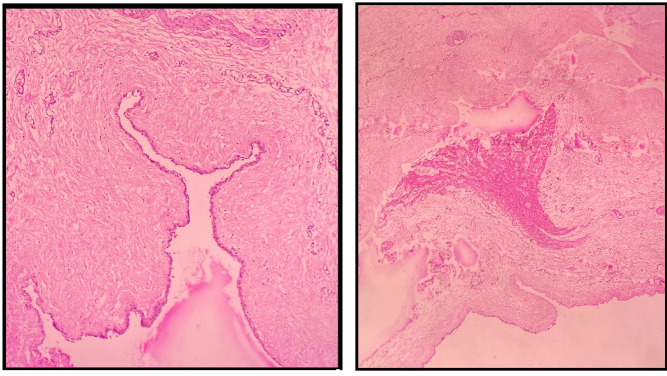
Histopathological images showing a single layer of mesothelial cells and blood and lymphatic vessels, smooth muscle cells, lymphocyte infiltration, and fibrosis.

Histopathology revealed a single layer of mesothelial cells. In addition to blood and lymphatic vessels, smooth muscle cells, lymphocyte infiltration, and fibrosis were observed (Figure 3). The follow-up echocardiogram after 2 months did not show any abnormalities and the patient is clinically stable with no symptoms.

## COMMENT

Pericardial cysts are uncommon benign tumors, which account for 7% of all mediastinal tumors with an incidence of 1:100,000^[1-3]^. Most are asymptomatic and found incidentally on chest radiographs. Seventy percent are located in the right cardiophrenic angle, 22% are in the left, and the remainder are in the anterior or posterior mediastinum^[4]^. It is usually composed of a single layer of flat or cuboidal mesothelium containing a clear, yellowish fluid^[1,5]^.

Epicardial cysts are much rarer than pericardial cysts, and their surgical management has seldom been reported. The surgical indications are when the cyst becomes symptomatic, infected or possibly malignant, grows progressively and compresses important adjacent structures^[2,6]^.

Herein, we report our experience with a posteroinferior epicardial cyst that was successfully resected with on-pump surgery. In our patient, excision was complicated because the cyst was in close proximity to the posterior descending artery. Resection followed by adequate evaluation and reinforcement of the thinned ventricular wall could not be accomplished without CPB. To avoid injury to the artery, we decided to operate with CPB support. CPB may be required when the cysts compress surrounding structures or erode the ventricular wall and coronaries^[2,3,7]^We suggest that the surgeon must be aware of the possible need for CPB in such situations.

Preoperative differential diagnosis between a pericardial cyst and an epicardial cyst is challenging because they cannot be differentiated on computed tomography (CT)^[5]^. Although MRI is useful to differentiate cysts from other cardiac lesions, it also cannot differentiate between pericardial and epicardial cysts. This should broaden the spectrum of the potential appearance of differentials such as epicardial masses. The role of cardiac magnetic resonance in tissue characterization to avoid erroneous preoperative diagnosis may have diagnostic importance.

Coronary CT or angiography might be considered especially with regard to the degree of adhesions and location of the coronary vessels. However, it may not always exclude the possibility of coronary involvement because some literature reported epicardial cysts with coronary vessel involvement and need for CPB to resect those cysts despite having negative findings^[1,2]^.

Video-assisted thoracoscopic surgery/thoracotomy may be the first option but when invasion of important structures, including the left ventricle and coronary artery is suspected, median sternotomy with CPB should be considered. Off-pump resection may be indicated for uncomplicated cysts^[8]^.

In our case, we were concerned about adhesions and proximity of the cyst to surrounding structures, thus the less invasiveness of the thoracotomy was not considered beneficial enough to outweigh the risks associated with inadmissible complications. Steady progression of the pathology and inflammation seem to be the reason for adhesions between the cyst and the pericardium. Surgical resection in CPB may be a better option when the cyst lies near important structures/erosion of the ventricular wall.

The primary take-away lesson from our reported incidence is that, despite detailed preoperative investigation and assessment, misdiagnosis of cardiac masses cannot be completely ruled out and erratically may completely simulate another condition which can change the therapeutic approach. In such a situation, the surgeon should act patiently and should prefer an approach which can be best for the patient.

**Table:** 

Authors' roles & responsibilities	
KG	Drafting the work or revising it critically for important intellectual content; final approval of the version to be published
DS	Drafting the work or revising it critically for important intellectual content; final approval of the version to be published
DN	Agreement to be accountable for all aspects of the work in ensuring that questions related to the accuracy or integrity of any part of the work are appropriately investigated and resolved; final approval of the version to be published
AC	Agreement to be accountable for all aspects of the work in ensuring that questions related to the accuracy or integrity of any part of the work are appropriately investigated and resolved; final approval of the version to be published
CS	Agreement to be accountable for all aspects of the work in ensuring that questions related to the accuracy or integrity of any part of the work are appropriately investigated and resolved; final approval of the version to be published
